# Diabetes

**Published:** 1898-03

**Authors:** 


### DIABETES.

Dr. William A. Thompson in a discussion in the Academy of Medicine, N. Y., concerning diabetes said:

He did not believe in physiological albuminuria or glycosuria, for he had repeatedly noted, in records extending over many years in private practice, that a very slight glycosuria had eventually developed into diabetes and Bright's disease respectively. Glycosuria was the first step toward diabetes, and the degree of severity varied very greatly in different individuals. Our clinical experience taught us that there was a very great difference in the disease depending upon certain factors. Foremost among these he would put age. The younger the person, the worse the prognosis. The only cases in which he was not very much disturbed by finding glycosuria were those of silvery-headed men and women. Those who became diabetic under twenty years of age usually exhibited a very uncontrollable form of the disease. He was firmly of the opinion that it was not the sugar that caused the fatal explosions of diabetes any more than it was the urea in Bright's disease that gave rise to the symptoms of uræmia, but that certain changes occurred in the sugar and allied bodies through the operation of toxins. These toxins were responsible for the development of acetonuria. He was always alarmed when a diabetic patient had a sweetish breath. Another important and closely related clinical fact was the disappearance of the urinary pigments. We had much yet to learn regarding the significance of pale urine in both diabetes and Bright's disease.

Opiates should be the last resort. He had always maintained that opium should be the very last resort in diabetes instead of being the sheet anchor, as many physicians seemed to believe. The preparations of opium should be used in diabetes, not as curative agents, but rather as a means of smoothing the path to the grave. His treatment of diabetes consisted in the systematic use of intestinal antiseptics, and he was especially strenuous on this point if he feared the supervention of coma; he also believed in the administration of arsenic, cod-liver oil, and iron, and sometimes of phosphorus and alkalies. He maintained that these agents were curative in a certain proportion of cases, particularly in those developing after forty years of age. He was always pleased to learn that a diabetic patient was gouty, because this materially improved the prognosis.

Diet should not be too exclusive. He was, however, in hearty accord with the reader of the paper regarding the matter of diet. If under a diet containing carbohydrates we found that the patient was not losing weight or developing special diabetic symptoms, no matter if the sugar still persisted, we were justified in keeping up such a diet. He had seen harm done by a too rigid exclusion of carbohydrates from the diet of diabetics. Bread could be taken in the form of very thin slices, so thoroughly browned as to be more than toasted. In his experience potatoes had not been nearly so injurious to diabetes as the cereals. He would much rather have a diabetic take a moderate amount of potato than the special diabetic breads. The question of prognosis should be determined by an experiment to ascertain the extent to which the sugar diminishes under a rigid exclusion of carbohydrates for a period of three days. If the proportion of sugar remained relatively high, the prognosis was certainly far more serious than when the reduction in the quantity was more prompt.

Premonitory symptoms of diabetes: Regarding the early symptoms of diabetes, the speaker said that the great majority of diabetics had sugar in the urine only intermittently at the outset. For this reason the urine should be repeatedly examined if a patient exhibited a decided form of nervous dyspepsia, or a condition of nervousness formerly unknown to the individual, or a tendency to pharyngeal catarrh, or when there was a tendency to cramps in the calves of the legs in the early morning. This last symptom was also common in gout and in chronic endarteritis, but when these conditions could be excluded the symptom was one strongly presumptive of diabetes. Under such circumstances the physician should examine both the morning and the evening urine for several days.—*Charlotte Medical Journal.*

